# Covalent Proteins as Targeted Radionuclide Therapies Enhance Antitumor Effects

**DOI:** 10.1021/acscentsci.3c00288

**Published:** 2023-06-07

**Authors:** Paul C. Klauser, Shalini Chopra, Li Cao, Kondapa Naidu Bobba, Bingchen Yu, Youngho Seo, Emily Chan, Robert R. Flavell, Michael J. Evans, Lei Wang

**Affiliations:** † Department of Pharmaceutical Chemistry and the Cardiovascular Research Institute, University of California San Francisco, San Francisco, California 94158, United States; ‡ Helen Diller Family Comprehensive Cancer Center, University of California San Francisco, San Francisco, California 94158, United States; § Department of Radiology and Biomedical Imaging, University of California San Francisco, San Francisco, California 94158, United States; ∥ Department of Pathology, University of California San Francisco, San Francisco, California 94158, United States

## Abstract

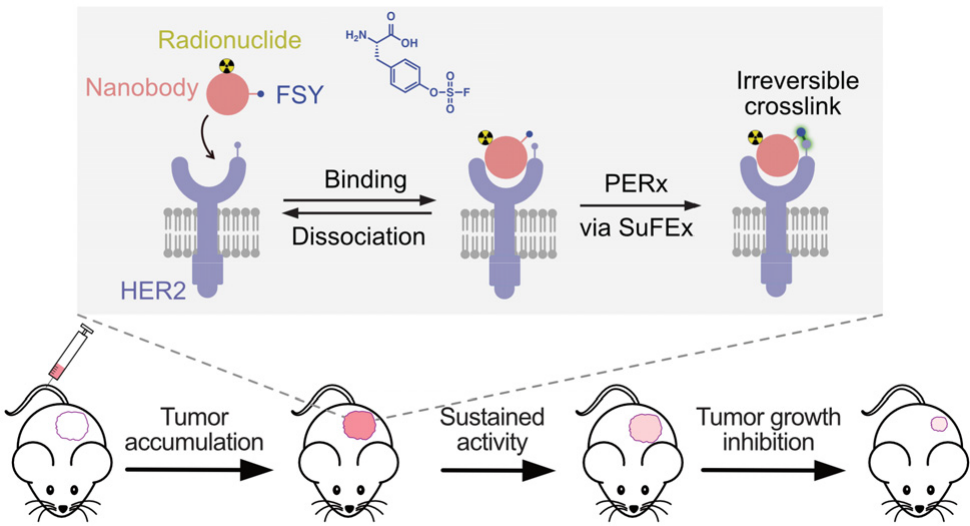

Molecularly targeted radionuclide therapies (TRTs) struggle with balancing efficacy and safety, as current strategies to increase tumor absorption often alter drug pharmacokinetics to prolong circulation and normal tissue irradiation. Here we report the first covalent protein TRT, which, through reacting with the target irreversibly, increases radioactive dose to the tumor without altering the drug’s pharmacokinetic profile or normal tissue biodistribution. Through genetic code expansion, we engineered a latent bioreactive amino acid into a nanobody, which binds to its target protein and forms a covalent linkage via the proximity-enabled reactivity, cross-linking the target irreversibly *in vitro*, on cancer cells, and on tumors *in vivo*. The radiolabeled covalent nanobody markedly increases radioisotope levels in tumors and extends tumor residence time while maintaining rapid systemic clearance. Furthermore, the covalent nanobody conjugated to the α-emitter actinium-225 inhibits tumor growth more effectively than the noncovalent nanobody without causing tissue toxicity. Shifting the protein-based TRT from noncovalent to covalent mode, this chemical strategy improves tumor responses to TRTs and can be readily scaled to diverse protein radiopharmaceuticals engaging broad tumor targets.

## Abstract 2

Through binding to target irreversibly, covalent nanobodies increase radioactive dose to the tumor without impairing normal tissues, inhibiting tumor growth more effectively than conventional nanobodies.

## Introduction

Molecularly targeted radionuclide therapies (TRTs) are a class of systemically administered, isotopically labeled drugs designed to concentrate ionizing radiation to all tumors in the body simultaneously.^1^ After localizing to tumors, these drugs exploit cancer’s well-known vulnerability to ionizing radiation by producing a continuous source of local radioactive emissions within the tumor to trigger severe and irreparable genetic damage. Since the approval of radioactive iodine for the treatment of well-differentiated thyroid cancer in the 1950s, TRT has found a place in standard of care as a safe alternative to external beam ionizing radiation for patients with targetable cancers including widely metastatic diseases.

Although TRT is a venerable treatment strategy for cancer, only within the past three decades has the nuclear medicine community developed new therapies for other cancer types that recapitulate the success of radioiodine.^1,2^ Indeed, TRT is experiencing a clinical renaissance, with several recent FDA approvals to treat metastatic castration-resistant prostate cancer (Pluvicto), neuroendocrine tumors (Lutathera), pheochromocytoma and paraganglioma (Azedra), and osseous metastases (Xofigo). Driving this renaissance has been the prioritization of low molecular weight (MW) TRTs, and particularly small molecule radioligands that rapidly exit the bloodstream to minimize host toxicity yet are still effective antitumor agents by binding highly overexpressed cancer proteins. This transition was motivated by 30 years of largely discouraging prior clinical experiences with various high MW radiopharmaceuticals such as immunoglobulins. Indeed, while the long serum half-life (3–7 days) of immunoglobulins results in high levels of target engagement and tumoral absorbed doses, the prolonged residence in the blood and slow hepatobiliary clearance results in high radiation exposure to radiosensitive normal tissue compartments (e.g., bone marrow) that results in toxicity, thus narrowing or eliminating a therapeutic index.^2^ The evolution of radiopharmaceuticals targeting prostate-specific membrane antigen (PSMA) stands out as an instructive case study on the tension between efficacy and safety. While various radiolabeled forms of the IgG J591, including ^177^Lu-J591, stalled in clinical trials due to dose limiting toxicities, Pluvicto (^177^Lu-PSMA 617), a low MW radioligand with weaker affinity for PSMA and lower tumor uptake compared to J591, nevertheless achieved FDA approval for prostate cancer treatment in 2022 due in large part to its better safety profile.^3−5^

However, low MW radioligand therapies (RLTs) are rarely curative, and more generally, developing drugs that fit the RLT paradigm is challenging. First, as the drug is rapidly exiting the body, to deliver sufficient dose to tumors, the field is limited to the small minority of highly overexpressed proteins in cancer that can extract sufficient radioligand from circulation. Indeed, prominent RLT drug targets like PSMA, somatostatin receptor type 2, fibroblast activated protein alpha (FAPα), carbonic anhydrase 9, and the bombesin receptor are all highly overexpressed on cancer cells (>10^5^ receptors per cell). Second, ligand/receptor complexes are intrinsically unstable in biology and subject to dissociation or degradation after endocytosis, reducing the effective radiation dose. Indeed, longitudinal PET studies in patients have shown that RLTs begin clearing from tumors within 96 h, and in some extreme cases (e.g., FAPI PET), the radioisotope washes out entirely from the tumor within a few hours.^6−9^ As leading therapeutic radioisotopes like lutetium-177 (^177^Lu) and actinium-225 (^225^Ac) have half-lives that span many days to even weeks, increasing their residence time in the tumor will likely confer more durable antitumor effects. Some investigators have approached this challenge by incorporating hydrophobic binding groups onto the scaffold of RLTs to encourage low affinity interactions with abundant serum proteins like albumin.^10,11^ While animal studies have shown that this strategy increases RLT uptake in tumors and subsequent tumor responses, a prolonged serum half-life increases irradiation to normal tissues and may incur toxicities. Other investigators have devised antibody pretargeting, wherein they administer a nonradioactive modified antibody followed by a radioligand that binds the antibody through noncovalent interactions or bioorthogonal chemistry.^12,13^ This strategy circumvents the slow pharmacokinetics while delivering a high dose of radiation to the cancer target. However, requiring two separate agents and a delayed delivery of the radioligand increases the complexity of the treatment. The ultimate clinical utility of these strategies remains to be determined.

**Figure 1:**
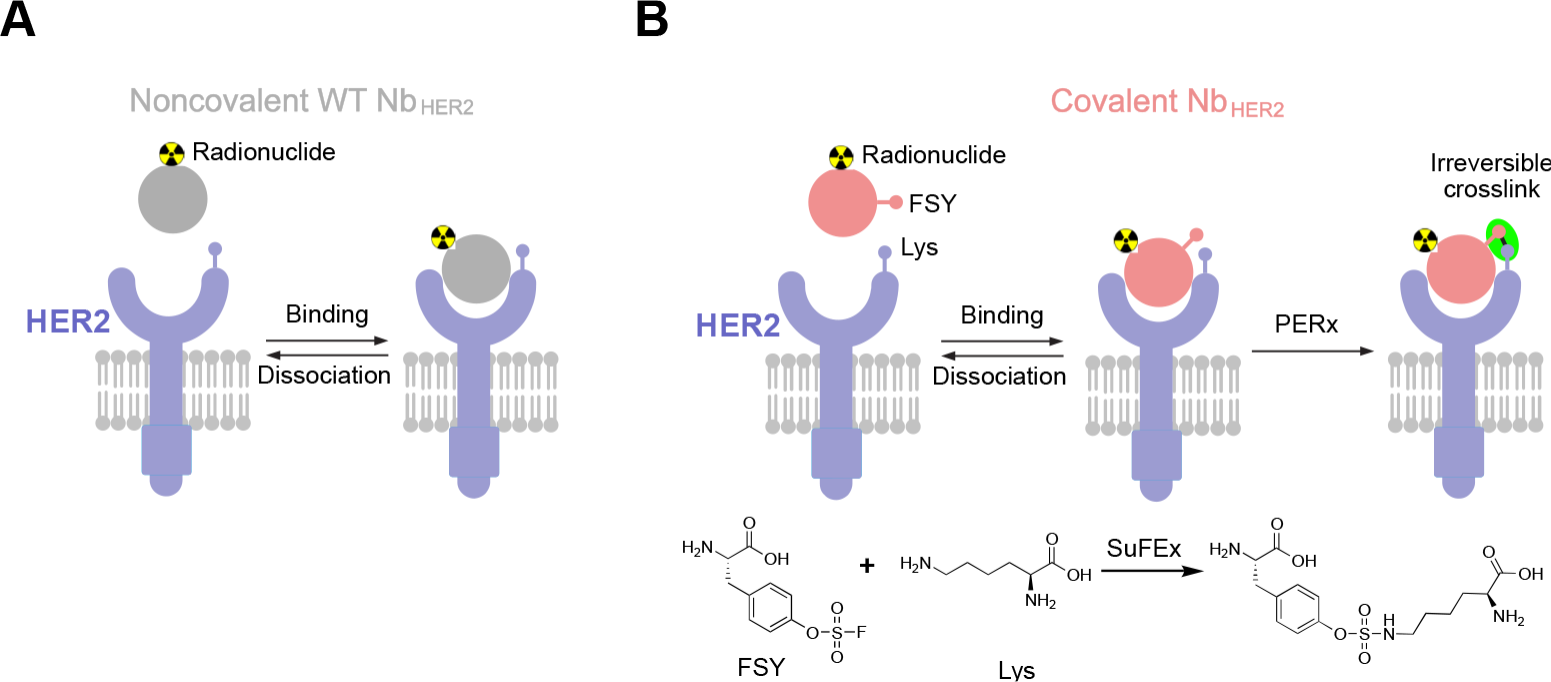
Covalent protein radiopharmaceuticals to enhance efficacy and safety for TRT. A schematic comparison of the noncovalent WT Nb_HER2_ (A) and the covalent Nb_HER2_ (B) in targeted delivery of radionuclide to HER2-expressing cancer cells. The noncovalent Nb_HER2_ binds HER2 reversibly allowing dissociation. In contrast, when the covalent Nb_HER2_ binds to HER2, the latent bioreactive Uaa FSY reacts with Lys through proximity-enabled SuFEx reaction, resulting in irreversible cross-linking of Nb_HER2_ with HER2 and persistent tumoral retention of the attached radionuclide.

An ideal radiopharmaceutical would have several characteristics, including high specificity, short blood and normal tissue residence time, and high tumor retention. Rather than trying to increase tumoral uptake of the TRT by manipulating serum half-life, we hypothesized that installing covalent reactivity in the TRT could be a strategy to lengthen the tumoral residence time without significantly altering time in circulation. While covalent reactivity has been installed on low MW radioligands,^14,15^ no covalent protein radiopharmaceutical has been developed for imaging and therapy. Here, we report the development of covalent protein radiopharmaceuticals that leverage proximity-enabled reactivity to bind target irreversibly. We generated a radiolabeled covalent nanobody that bound the human epidermal growth factor receptor 2 (HER2) irreversibly *in vitro* and on cancer cell surfaces (Figure 1). Using positron emission tomography (PET), we showed that the covalent nanobody attained highly specific and longer tumor accumulation *in vivo* than the wild-type nanobody. We further demonstrated that the ^225^Ac-labeled covalent nanobody more effectively inhibited the growth of HER2-expressing tumors in mice compared to the wild-type nanobody. We showed that not only did the ^225^Ac-labeled covalent nanobody inhibit tumor growth at a greater level than the noncovalent counterpart, it also showed no toxicity in key tissues such as the heart, liver, kidneys, or bone marrow. This covalent protein radiopharmaceutical strategy highlights the potential to employ covalent chemistry on proteins *in vivo* and to shift the protein-based TRT from noncovalent to covalent binding mode for precision medicine.

## Results

### Developing Covalent Nanobody Radiopharmaceuticals via Proximity-Enabled Reactivity

We envisioned that a covalent protein radiopharmaceutical would be fast-clearing in circulation but achieve persistent tumor residence through binding the cancer target specifically and irreversibly. However, native proteins and engineered protein binders such as nanobodies and antibodies generally bind to their targets through reversible noncovalent interactions.^16^ To break this natural barrier, we recently reported a Proximity-Enabled Reactive Therapeutics (PERx) strategy to generate covalent protein drugs.^17,18^ Through genetic code expansion,^19^ a latent bioreactive unnatural amino acid (Uaa) was incorporated into the protein drug, which selectively forms a covalent linkage with a proximal natural residue of the target protein only upon drug-target interaction, resulting in the irreversible binding of the protein drug to its target.^16,18,20^ We have demonstrated that PERx-enabled covalent protein drugs showed drastically higher potency in cancer immunotherapy and in neutralization of SARS-CoV-2 over the noncovalent wild-type proteins.^18,21^ Aside from initial success in increasing drug potency, whether PERx-enabled biocompatible covalent chemistry can advance protein therapeutics via new mechanisms awaits exploration.

Nanobodies have small molecule weight (∼15 kDa) for efficient tumor penetrance and rapid clearance from circulation, are generally heat stable and easy to produce in bacteria, can be humanized to minimize potential immunogenicity, and can be readily evolved to bind various targets in high specificity. Our strategy for developing covalent protein radiopharmaceuticals thus started with genetically incorporating a latent bioreactive Uaa into the nanobody followed with radioisotope labeling. We recently genetically incorporated a latent bioreactive Uaa, fluorosulfate-l-tyrosine (FSY), which is stable in cells and reacts with Lys, His, or Tyr residue on proteins through Sulfur Fluoride Exchange (SuFEx) click chemistry^22^ only when the two residues are in close proximity.^23,24^ We therefore decided to incorporate FSY into Nb_HER2_,^25^ a nanobody specific for HER2, to generate a covalent nanobody as the delivery vehicle for radionuclides for PET imaging and TRT (Figure 1B). HER2 gene amplification and overexpression occurs in a number of different cancers including breast, stomach, ovarian, kidney, prostate, salivary glands, colon, urinary, and lung.^26^ To image HER2-positive cancer, PET has been the modality of choice for the clinic due to its high spatial resolution and sensitivity.^27^ Only upon Nb_HER2_ binding to HER2 would FSY selectively react with a target residue of HER2 via proximity-enabled SuFEx reactivity and thus cross-link them irreversibly (Figure 1B). The conventional nanobody binds in noncovalent mode and is in dynamic association and dissociation with HER2, which will be cleared from HER2-expressing cells; in contrast, the covalent nanobody would permanently bind to HER2 and thus enhance the specific accumulation of the attached radionuclide to HER2 expressing cells. At nontarget sites, the covalent nanobody will not generate such covalent cross-link and, thus, is quickly cleared as the conventional nanobody to minimize background.

### Genetically Encoding FSY to Generate Nanobody Targeting HER2 Covalently

**Figure 2:**
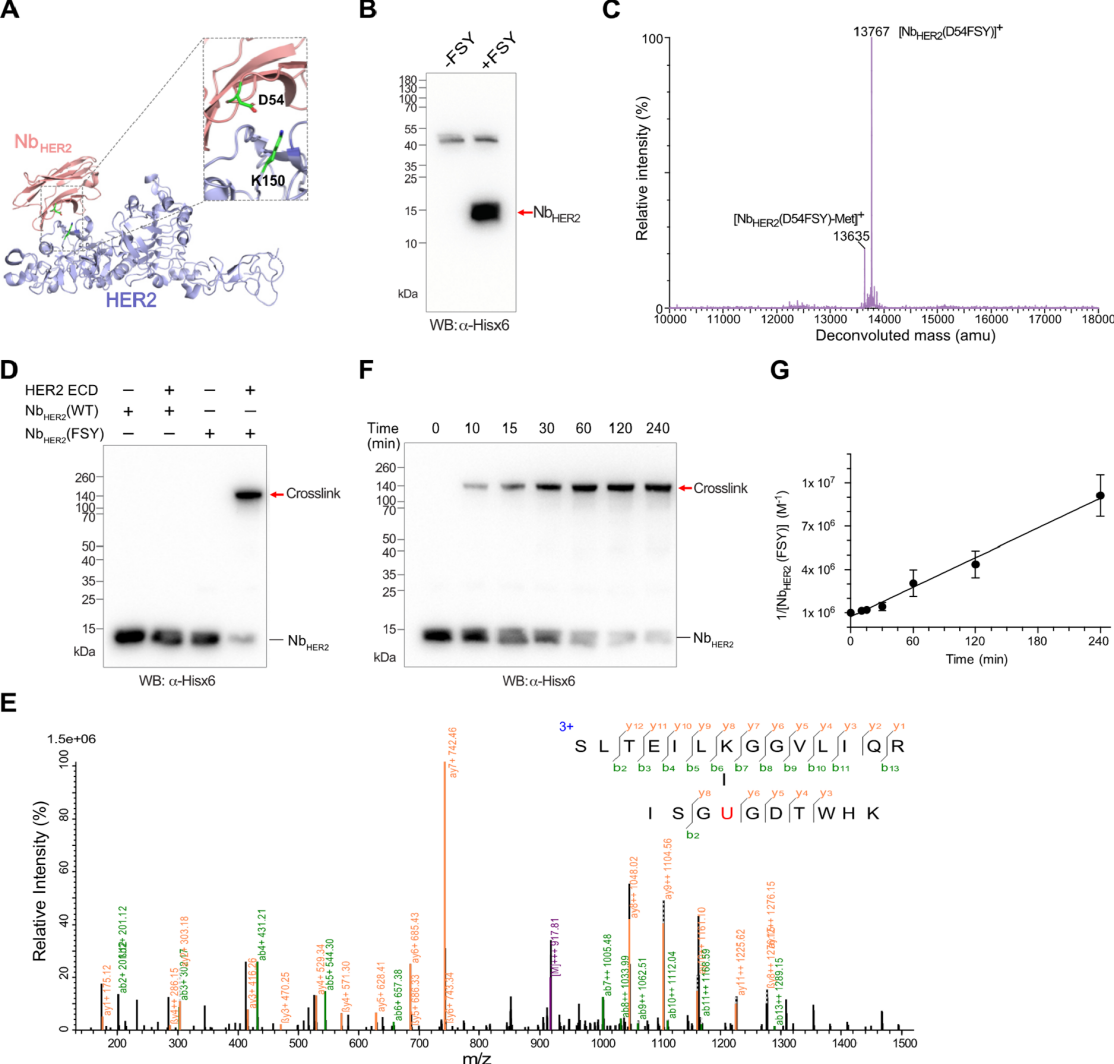
Genetically encoding FSY in Nb_HER2_ to covalently cross-link HER2 irreversibly *in vitro*. (A) Crystal structure of Nb_HER2_ bound to HER2 ECD (PDB: 5MY6), showing the FSY incorporation site (D54) and the proximal target residue (K150) in HER2. (B) Western blot analysis of Nb_HER2_(FSY) production in *E. coli* with and without 1 mM FSY in growth media. A His6x tag was appended at the C-terminus of Nb_HER2_ for detection. (C) Mass spectrum of the intact Nb_HER2_(FSY) protein confirming FSY incorporation at position 54 in high fidelity. (D) Nb_HER2_(FSY), but not Nb_HER2_(WT), cross-linked with HER2 ECD *in vitro*. Indicated proteins were incubated at 37 °C for 4 h followed with Western blot analysis. (E) Tandem mass spectrum of Nb_HER2_(FSY) incubation with HER2 ECD confirmed that FSY (represented by U) of Nb_HER2_(FSY) cross-linked with Lys150 of HER2 as designed. (F) Cross-linking of Nb_HER2_(FSY) to HER2 ECD occurred efficiently at 10 min and increased with time. (G) Kinetics of Nb_HER2_(FSY) cross-linking with HER2 ECD. Nb_HER2_(FSY) concentrations in (F) were measured with densitometry and 1/[Nb_HER2_(FSY)] was plotted against time. Linear regression of the data yielded a second-order rate constant of 34154 ± 1921 M^–1^min^–1^ (mean ± s.d.). Error bars represent s.d., *n* = 3 independent experiments.

We first generated a covalent Nb_HER2_ to irreversibly cross-link HER2 *in vitro*. Based on the structure of Nb_HER2_ in complex with HER2 extracellular domain (ECD),^25,28^ we chose Asp54 on Nb_HER2_ as a potential site for FSY incorporation to target Lys150 in proximity on HER2 ECD (Figure 2A). The Nb_HER2_(FSY) mutant protein was produced in *E. coli* through expressing the Nb_HER2_ gene containing a TAG stop codon at site 54 together with the genes for tRNA^Pyl^-FSYRS,^24^ which incorporates FSY in response to TAG. Western blot analysis of the cell lysate showed that full-length Nb_HER2_ was produced only when 1 mM of FSY was added to the growth media (Figure 2B), suggesting FSY incorporation at the TAG site. The Nb_HER2_(FSY) protein was purified with affinity chromatography in the yield of 0.5 mg/L. To further evaluate the fidelity of FSY incorporation, the purified Nb_HER2_(FSY) protein was analyzed by electrospray ionization time-of-flight mass spectrometry (Figure 2C and Figure S1 for WT Nb_HER2_). A peak was observed at 13767 Da, which corresponds to intact Nb_HER2_ containing a single FSY residue at position 54 (expected [M + H]^+^ = 13767 Da). A second peak measured at 13635 Da corresponds to Nb_HER2_(FSY) lacking the initiating Met (expected [M – Met + H]^+^ = 13635 Da), which is expected for proteins expressed in *E. coli* cells. No peaks corresponding to proteins containing any other amino acids at position 54 were observed, confirming high fidelity of FSY incorporation in Nb_HER2_. To check if FSY incorporation affected Nb_HER2_ binding to HER2, we measured the association of Nb_HER2_ with HER2 using biolayer interferometry (Figure S2). HER2 was incubated with varying concentrations of Nb_HER2_(WT) or Nb_HER2_(FSY) for 90 s. The association rate constant *k*_on_ was measured to be (1.21 ± 0.01) × 10^5^ M^–1^ s^–1^ for Nb_HER2_(WT) and (1.15 ± 0.02) × 10^5^ M^–1^ s^–1^ for Nb_HER2_(FSY), suggesting a similar association rate of Nb_HER2_(WT) and Nb_HER2_(FSY) with HER2.

To test if Nb_HER2_(FSY) could covalently cross-link the HER2 ECD, we incubated Nb_HER2_(WT) or Nb_HER2_(FSY) with and without HER2 ECD at 37 °C for 4 h followed with Western blot analysis. A covalent complex was detected only when HER2 ECD was incubated with Nb_HER2_(FSY) (Figure 2D), indicating that the cross-linking was dependent on FSY reactivity as designed. To determine which residue of HER2 was cross-linked by FSY, we trypsin digested the cross-linked Nb_HER2_(FSY)-HER2 and analyzed the digested sample with tandem mass spectrometry in high resolution. The cross-linked peptide was identified, and a series of b and y ions of the cross-linked peptide unambiguously indicated that FSY54 in Nb_HER2_ reacted with Lys150 in HER2 (Figure 2E). No other residues of HER2 were found reacted with FSY, indicating that Nb_HER2_(FSY) covalently targeted HER2 on Lys150 as predicted from the crystal structure in a highly specific manner. To further evaluate the kinetics of covalent complex formation, Nb_HER2_(FSY) was incubated with HER2 ECD for different time duration and analyzed with Western blot (Figure 2F). Cross-linking was detected as soon as 10 min of incubation at 37 °C, and a second-order rate constant of 34154 ± 1921 M^–1^min^–1^ was measured (Figure 2G), indicating that Nb_HER2_(FSY) rapidly and efficiently cross-linked the HER2 ECD *in vitro*.

### Covalent Nanobody Nb_HER2_(FSY) Irreversibly Cross-links Native HER2 on Cancer Cells and on Tumor *In Vivo*

**Figure 3:**
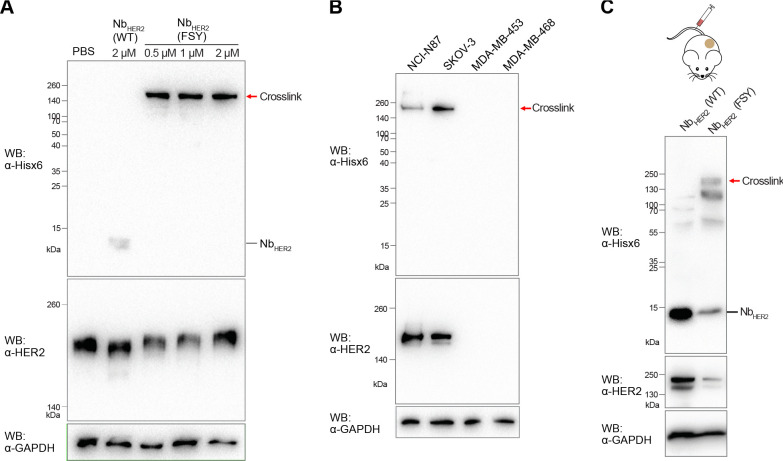
Nb_HER2_(FSY) covalently cross-linked native HER2 on cancer cells and on tumor *in vivo*. (A) Nb_HER2_(FSY) covalently cross-linked HER2 on NCI-N87 cell surface. Nb_HER2_ proteins were incubated with NCI-N87 cells for 3 h followed with Western blot analysis. (B) Cross-linking of Nb_HER2_(FSY) with cancer cells were HER2 specific. Cross-linking occurred only on NCI-N87 and SK-OV-3 cells, which have detectable HER2 expression. (C) Nb_HER2_(FSY) covalently cross-linked HER2 on NCI-N87 tumor *in vivo*. Nb_HER2_(FSY) or Nb_HER2_(WT)was injected into mice xenografted with HER2-expressing NCI-N87 tumor. After 6 h postinjection, the tumor was excised and homogenized, followed with Western blot analysis.

We next tested if Nb_HER2_(FSY) could covalently cross-link full-length native HER2 receptor on the cell surface of NCI-N87, a HER2-positive gastric cancer cell line. We treated NCI-N87 cells with different concentrations of Nb_HER2_(FSY) and compared to PBS and Nb_HER2_(WT). Cells were then lysed and analyzed with Western blot (Figure 3A). PBS or Nb_HER2_(WT) treated cells did not show any cross-linking of HER2, whereas Nb_HER2_(FSY) treated cells all exhibited a covalent complex of HER2 with Nb_HER2_(FSY). In addition, to determine if cell surface cross-linking was HER2-dependent, we treated additional cell lines with varying expression level of HER2. NCI-N87 and SK-OV-3 (ovarian cancer) both have high HER2 expression, while MDA-MB-453 and MDA-MB-468 (breast cancer) both have undetectable HER2 expression. Covalent HER2 cross-linking by Nb_HER2_(FSY) was detected on NCI-N87 and SK-OV-3 cells but not on MDA-MB-453 and MDA-MB-468 cells (Figure 3B). Moreover, except with HER2, no other cross-linking bands were detected for Nb_HER2_ in all four tested cell lines, suggesting that Nb_HER2_(FSY) was highly selective in cross-linking the HER2 receptor on cell surface.

We further tested whether Nb_HER2_(FSY) could cross-link HER2 on tumor *in vivo*. Nb_HER2_(WT) or Nb_HER2_(FSY) was delivered via intravenous tail vein injection into mouse xenografted with HER2-expressing NCI-N87 tumor. The tumor was dissected 6 h postinjection, homogenized and immunoblotted to detect cross-linking. Nb_HER2_(WT) did not yield any cross-linking with HER2, whereas Nb_HER2_(FSY) showed apparent cross-linking with HER2 (Figure 3C). Taken together, the *in vitro*, on-cell and on-tumor cross-linking assays indicate that Nb_HER2_(FSY) was able to bind to the HER2 receptor selectively, efficiently, and irreversibly.

### Covalent ^124^I-Nb_HER2_(FSY) Enhances Tumor Retention and PET Imaging in Mice

**Figure 4:**
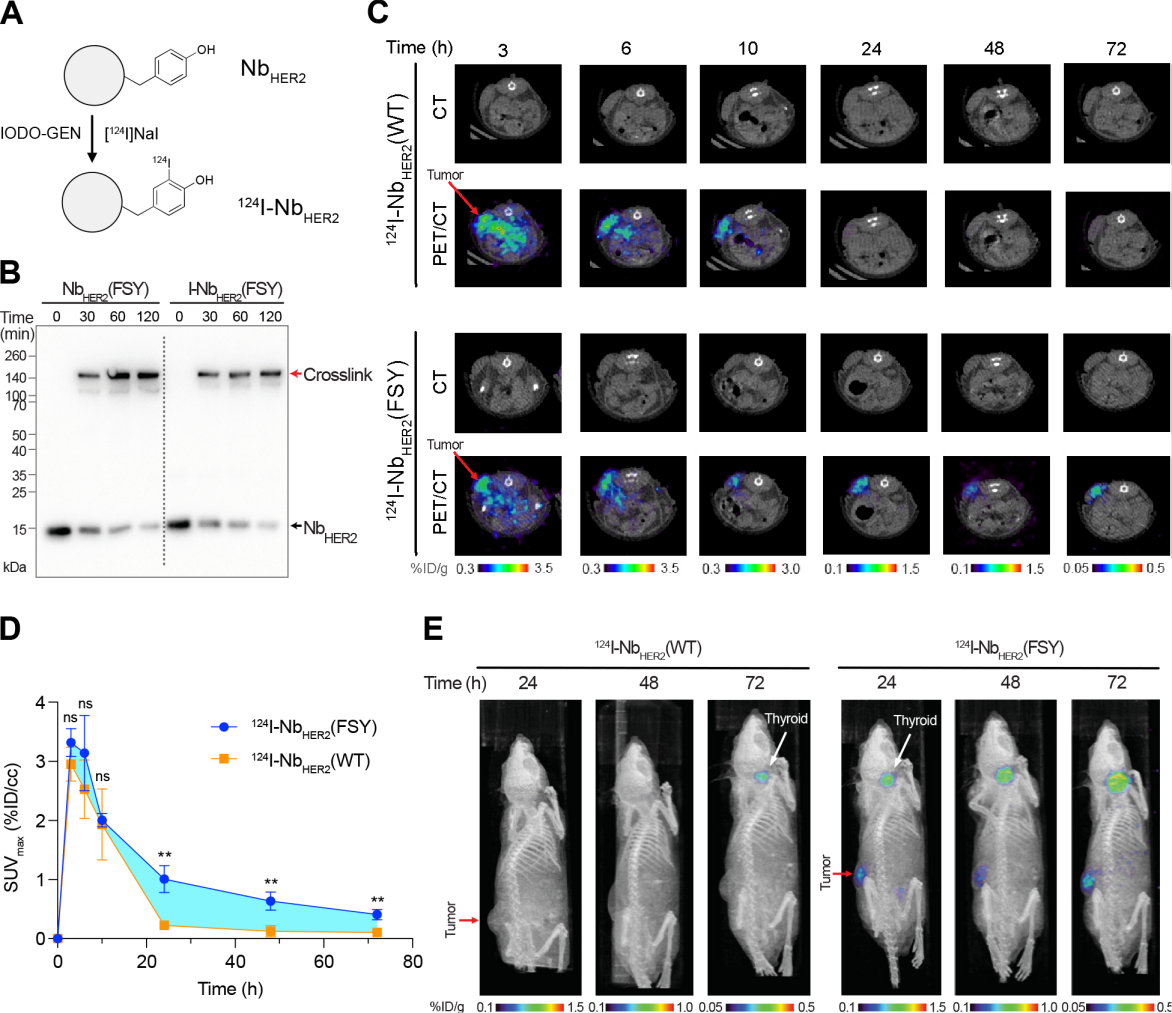
Radiolabeled covalent nanobody ^124^I-Nb_HER2_(FSY) prolonged tumor retention, increased tumor accumulation and exhibited low background in mice. (A) Schematic procedures to radiolabel WT and covalent Nb_HER2_ with ^124^I by IODO-GEN. Tyrosine is usually labeled at the ortho position with mono- or di-iodination. (B) Iodine labeling did not impair Nb_HER2_(FSY) cross-linking with HER2. The cold NaI labeled product I-Nb_HER2_(FSY) or the unlabeled Nb_HER2_(FSY) was incubated with HER2 ECD for cross-linking, followed with Western blot analysis. (C) The covalent ^124^I-Nb_HER2_(FSY) enabled specific and sustained tumor accumulation of ^124^I. Tumors were clearly detectable 24–72 h postinjection for ^124^I-Nb_HER2_(FSY) but not ^124^I-Nb_HER2_(WT). Representative decay-corrected PET images of mice xenografted with HER2-expressing NCI-N87 tumor and injected with either ^124^I-Nb_HER2_(WT) or ^124^I-Nb_HER2_(FSY) are shown. The transverse images of mice were taken at 3–72 h postinjection. Color bars indicate percent injected dose per gram (%ID/g). (D) The covalent ^124^I-Nb_HER2_(FSY) significantly enhanced tumor accumulation of ^124^I than ^124^I-Nb_HER2_(WT). The standardized uptake value (SUV) of ^124^I in tumor was quantified in percent injected dose per cm^3^ (%ID/cc) and plotted with postinjection time. The increase in tumor uptake by ^124^I-Nb_HER2_(FSY) over ^124^I-Nb_HER2_(WT) is highlighted in cyan. Error bars represent s.d.; *n* = 3 mice for ^124^I-Nb_HER2_(WT) injection; *n* = 4 mice for ^124^I-Nb_HER2_(FSY) injection; ns, not significant; ** *p* < 0.01; Student’s *t* test for statistical analysis. (E) The covalent ^124^I-Nb_HER2_(FSY) enabled clear imaging of tumor distinct from the background. 3D PET image reconstruction of mice 24–72 h postinjection of ^124^I-Nb_HER2_(WT) or ^124^I-Nb_HER2_(FSY) are shown. Color bars indicate %ID/g. *n* = 3 mice for ^124^I-Nb_HER2_(WT) injection; *n* = 4 mice for ^124^I-Nb_HER2_(FSY) injection.

To assess if Nb_HER2_(FSY) could enhance tumor accumulation and target-to-background ratio, we radiolabeled Nb_HER2_(WT) and Nb_HER2_(FSY) and monitored the resultant radiopharmaceuticals in xenografted mice through microPET/CT imaging. Nb_HER2_(WT) and Nb_HER2_(FSY) were labeled with iodine-124 (^124^I) using [^124^I]NaI and the established iodination reagent IODO-GEN (Figure 4A).^29^^124^I is a positron emitter with a long half-life (*t*_1/2_ ∼ 4.2 days) suitable for PET and pharmacokinetic studies.^30^ The radiochemical purity was 99.9% for ^124^I-Nb_HER2_(WT) and 95.2% for ^124^I-Nb_HER2_(FSY) (Figure S3). We also similarly labeled Nb_HER2_(FSY) with cold NaI and showed that iodine labeling did not impair the ability of Nb_HER2_(FSY) to covalently cross-link HER2 (Figure 4B). Next, male nude mice bearing subcutaneous NCI-N87 tumor were injected with ^124^I-Nb_HER2_(WT) or ^124^I-Nb_HER2_(FSY) intravenously. Both ^124^I-Nb_HER2_(WT) and ^124^I-Nb_HER2_(FSY) were coinjected with l-lysine to avoid peak catabolism in the kidneys for renal protection.^31,32^ To evaluate pharmacokinetics, blood clearance of ^124^I-Nb_HER2_(WT) and ^124^I-Nb_HER2_(FSY) was monitored using a dynamic PET acquisition for 90 min postinjection on a dedicated small animal microPET/CT. Both were cleared from blood circulation rapidly (Figure S4). The *t*_1/2_ for fast phase was measured 5.76 s for ^124^I-Nb_HER2_(WT) and 3.35 s for ^124^I-Nb_HER2_(FSY), suggesting that FSY incorporation did not prolong the desired rapid clearance of the radio-labeled nanobody.

The mice were subsequently imaged with microPET/CT. The radiotracer uptake in liver, kidney, thyroid, and skeletal muscle were qualitatively similar for ^124^I-Nb_HER2_(WT) and ^124^I-Nb_HER2_(FSY) (Figure S5), indicating that FSY incorporation did not significantly alter the biodistribution of the radiolabeled nanobody in normal organs lacking HER2. In contrast, a marked difference was detected on the tumor. From 3 to 10 h postinjection, the on-tumor activity showed similar levels between ^124^I-Nb_HER2_(WT) and ^124^I-Nb_HER2_(FSY) in the PET images (Figure 4C). However, a dramatic difference was observed from 24 to 72 h postinjection. At 24 h post injection, ^124^I-Nb_HER2_(WT) was no longer detectable in tumor, whereas ^124^I-Nb_HER2_(FSY) was clearly detectable in tumor from 24–72 h post injection. Quantification of tumoral uptake using region of interest analysis revealed that ^124^I-Nb_HER2_(FSY) had ∼4.5, 5, and 4-fold of activity over ^124^I-Nb_HER2_(WT) at 24, 48, and 72 h postinjection, respectively (Figure 4D). The total radiation, quantified by area under the curve (AUC), was 78 ± 4 for ^124^I-Nb_HER2_(FSY) and 43 ± 4 for ^124^I-Nb_HER2_(WT), showing 81.4% more radiation accumulation to tumor by ^124^I-Nb_HER2_(FSY). Three-dimensional maximum intensity projections of the PET/CT data showed that, from 24 to 72 h postinjection, mice injected with ^124^I-Nb_HER2_(FSY) had the tumor distinctly visible and virtually no retention in normal tissues with the exception of the thyroid (Figure 4E). The thyroid was visible due to scavenging of free ^124^I anion that is known released by catabolism *in vivo*.^33^ Extended retention of ^124^I-Nb_HER2_(FSY) at the tumor site thus would result in the observed higher level of thyroid uptake than ^124^I-Nb_HER2_(WT). Collectively, these data show that the covalent nanobody dramatically improved tumoral retention of the labeled radionuclide without changing the pharmacokinetic profile.

### Covalent ^225^Ac-Nb_HER2_(FSY) Inhibits Tumor Growth in Mice

We next asked if the increase in tumoral retention of the covalent nanobody compared to the WT nanobody was sufficiently large to impact antitumor effects. To address this question, we prepared Nb_HER2_(WT) and Nb_HER2_(FSY) labeled with ^225^Ac, an emerging radioisotope that produces alpha emissions. We chose ^225^Ac because α-emitters are more effective antitumor agents due to their higher linear energy transfer properties compared to β-emitters like ^177^Lu,^34^ and the tumoral uptake levels of the nanobody would likely necessitate a potent payload. Moreover, ^225^Ac TRTs are under clinical investigation, and the early data suggest the radioisotope is well tolerated *in vivo*.^35,36^

**Figure 5:**
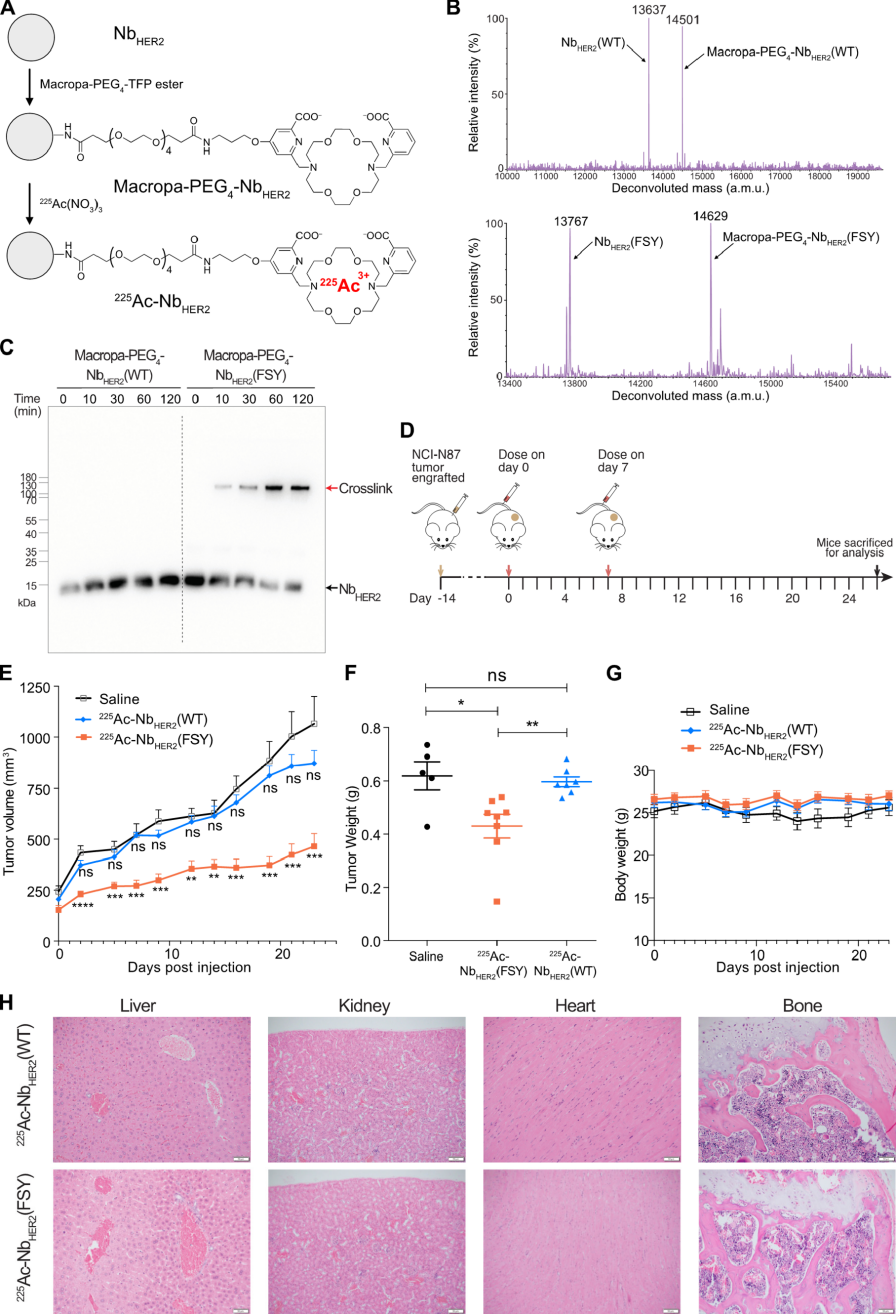
α-Emitter labeled covalent ^225^Ac-Nb_HER2_(FSY) inhibited tumor growth in mice without tissue toxicity. (A) Schematic procedures to radiolabel WT and covalent Nb_HER2_ with ^225^Ac. (B) Mass spectrometric analyses confirming successful conjugation of Macropa-PEG_4_ on Nb_HER2_(WT) (top panel) and Nb_HER2_(FSY) (bottom panel). (C) Western blot analysis confirming that Macropa-PEG_4_ labeling did not impair Nb_HER2_(FSY) cross-linking with HER2. Cross-linking of Macropa-PEG_4_-Nb_HER2_(FSY) to HER2 ECD occurred efficiently after 10 min incubation and increased with time, while no cross-linking was detected with Macropa-PEG_4_-Nb_HER2_(WT). (D) Experiment scheme for TRT of NCI-N87 tumor in mice. (E) Growth curves of engrafted NCI-N87 tumors indicate that ^225^Ac-Nb_HER2_(FSY) inhibited tumor growth, while ^225^Ac-Nb_HER2_(WT) did not. (F) Weight comparison of dissected tumors showing tumor weight reduction by ^225^Ac-Nb_HER2_(FSY) treatment. (G) Mice body weight remained stable over the course of the therapy study. For panels E–G, error bars represent SEM; *n* = 8 mice for ^225^Ac-Nb_HER2_(FSY) treatment group; *n* = 7 mice for ^225^Ac-Nb_HER2_(WT) treatment group; *n* = 5 mice for vehicle saline control. ns, not significant; *p < 0.05; ** *p* < 0.01; *** *p* < 0.001; **** *p* < 0.0001; Student’s *t* test for statistical analysis. (H) Representative microscopic images of hematoxylin and eosin stained liver, kidneys, heart, and bone marrow for both ^225^Ac-Nb_HER2_(WT) and ^225^Ac-Nb_HER2_(FSY) treatment groups. No abnormalities were detected in the tissues. Scale bar, 50 μm.

To prepare for the TRTs, Nb_HER2_(WT) and Nb_HER2_(FSY) were conjugated with Macropa-PEG_4_-TFP ester (Figure 5A). Macropa was chosen as the chelator, as recent data have shown that it chelates ^225^Ac efficiently.^37,38^ Mass spectrometric analysis of the conjugated samples confirmed that both Nb_HER2_(WT) and Nb_HER2_(FSY) were successfully conjugated with Macropa-PEG_4_, showing two peaks of approximately equal intensity corresponding to the unlabeled and singly labeled nanobody, respectively (Figure 5B). To ensure that the conjugation of Macropa-PEG_4_ did not affect the nanobody’s covalent cross-linking ability, we incubated Macropa-PEG_4_-Nb_HER2_(WT) or Macropa-PEG_4_-Nb_HER2_(FSY) with and without the HER2 ECD at 37 °C for up to 2 h and analyzed the samples via Western blot (Figure 5C). The Macropa-PEG_4_-Nb_HER2_(FSY) could still effectively cross-link HER2, suggesting that the Macropa-PEG_4_ label had not negatively impacted the covalency of our nanobody. Macropa-PEG_4_-Nb_HER2_(WT) and Macropa-PEG_4_-Nb_HER2_(FSY) were then radiolabeled with ^225^Ac, yielding ^225^Ac-Nb_HER2_(WT) and ^225^Ac-Nb_HER2_(FSY), respectively. The radiochemical purity was >95% for ^225^Ac-Nb_HER2_(WT) and ^225^Ac-Nb_HER2_(FSY) (Figure S6).

To evaluate TRT efficacy *in vivo*, we xenografted HER2-expressing NCI-N87 tumors subcutaneously in male athymic nu/nu mice and treated them twice with either ^225^Ac-Nb_HER2_(WT), ^225^Ac-Nb_HER2_(FSY), or saline via tail vein injection on day 0 and day 7 (Figure 5D). The mice received doses of ∼0.8 μCi at the same molar activity (0.67 μCi/pmol). Tumor growth was measured over 23 days via calipers, and the mice were euthanized on day 26. When compared with the saline control, while injection with ^225^Ac-Nb_HER2_(WT) showed no tumor growth inhibition, injection with the covalent nanobody ^225^Ac-Nb_HER2_(FSY) slowed down tumor growth significantly (Figure 5E). Endpoint analysis also showed that the tumor weight was significantly reduced when mice were treated with ^225^Ac-Nb_HER2_(FSY) but not with ^225^Ac-Nb_HER2_(WT) (Figure 5F). The body weight changes serve as a sensitive indicator of general health status. The weight of the mice in either of the three groups did not change significantly after treatment (Figure 5G), indicating no systemic toxicity. To further demonstrate that the radiolabeled nanobodies did not cause significant toxicity to organs, the liver, kidney, heart and bone marrow were treated with hematoxylin and eosin stains and examined by an independent pathologist for signs of abnormalities. Most ^225^Ac radiation-induced toxicity occurs at either the liver, kidney, or bone marrow. HER2-targeting drugs often cause cardiotoxicity,^39^ and therefore the heart was analyzed as well. No abnormalities were detected in any tissue samples from the groups treated with either ^225^Ac-Nb_HER2_(FSY) or ^225^Ac-Nb_HER2_(WT) (Figure 5H), suggesting no toxicity and systematic clearance of the radiolabeled nanobodies after treatment.

## Discussion

As we now understand TRT is a viable option to treat common and heterogeneous solid tumor types like metastatic castration-resistant prostate cancer, there is an urgent need to develop new strategies to maximize their antitumor activity. This is an unusual challenge, as many of the approaches used to attenuate the toxicity of other cancer therapeutics are likely not relevant to TRT. For example, prodrug masking, one of the most venerable approaches for restricting drug activity to tumors, is likely not possible for TRT given the continuous decay of the isotopic payload. It is also not evident whether common drug delivery strategies used to expand the therapeutic window for chemotherapies (e.g., liposome encapsulation) have any relevance to TRT, as the TRT mass dose is often so low that the ligand’s bioactivity rarely factors into its pharmacological profile. The mode of administration is also expected to be limited to intravenous, intra-arterial, or intratumoral routes, as rapid delivery to the tumor is essential to limit host toxicity. Thus, and quite tragically, the field has been stuck in a safety/efficacy dilemma wherein increasing tumor absorption of the TRT is best achieved by lengthening its serum half-life, which by necessity further increases radiation to normal tissue compartments.

Herein, we present the first chemical strategy to increase tumor absorption of a protein-based radiopharmaceutical without impacting its other pharmacokinetic properties. By genetically engineering the latent bioreactive Uaa FSY into the nanobody Nb_HER2_, we generated a covalent nanobody that specifically and irreversibly targeted HER2 via the PERx mechanism *in vitro*, on cancer cells, and on tumor *in vivo*. With radioisotope ^124^I labeling, the covalent nanobody enhanced radionuclide accumulation and showed prolonged residence in HER2-expressing tumors while still maintaining fast clearance from circulation in mice, which enabled exceptional contrast for tumor detection and low background activity in other tissues for molecular imaging in mice. When we labeled the same covalent nanobody with the potent α-emitter ^225^Ac, the resultant covalent ^225^Ac-Nb_HER2_(FSY) had a much higher antitumor efficacy targeting HER2-expressing tumors than the ^225^Ac-Nb_HER2_(WT) counterpart while having no detectable toxicity in normal tissues.

Leveraging fast-clearing proteins to bind target covalently, our method thus can enable a new class of radiopharmaceuticals for TRT to simultaneously achieve efficacy and safety. Existing protein radiopharmaceuticals bind their targets only through noncovalent interactions; our covalent protein radiopharmaceutical changes this paradigm and exploits the therapeutic benefits of covalency. The covalent binding is realized through proximity-enabled reactivity of the latent bioreactive Uaa, which safeguards the reaction to be highly specific between the covalent protein and its target.^18^ Indeed, off-target cross-linking is not detected *in vivo* in mice or in human serum.^18^ In this study, similar systemic clearance of radiolabeled Nb_HER2_(FSY) as Nb_HER2_(WT) and no tissue abnormalities both suggest no off-target covalent binding. The proximity-enabled reaction mechanism of our covalent protein radiopharmaceutical thus uniquely allows it to react and durably reside at the tumor site only, which is critical for the improved efficacy and safety. Indeed, a recent TRT study dosed 2.29 μCi of ^225^Ac-labeled noncovalent WT Nb_HER2_ in mice, which results in substantial inflammatory lesions in kidney.^40^ Our covalent Nb_HER2_(FSY) permitted a drastic lower dose of 0.8 μCi for tumor inhibition and did not cause tissue toxicities.

Our method can readily expand the repertoire of radiopharmaceuticals that work in the unique covalent mechanism to target a broad range of cancer-specific proteins with various expression levels. Radiopharmaceuticals approved for radionuclide therapy in oncology have used small-molecule, peptide or antibody as the delivery vehicle with caveats either in efficacy or safety.^1^ Through irreversible covalent binding, our method will enable the broad use of proteins with MW below the renal filtration threshold as the delivery vehicle. Aside from nanobody demonstrated herein, these proteins can be affibody,^18^ single-chain variable fragment, Fab,^41^ DARPins, *de novo* designed mini-binders, and so on, which can be readily developed with well-defined binding and selectivity against various antigens. Our method requires the incorporation of only a single latent bioreactive amino acid, and genetic incorporation of latent bioreactive Uaas into proteins can be carried out in both prokaryotic and eukaryotic cells,^42,43^ permitting the ready conversion of all these proteins into covalent proteins. In addition, through chemically synthesizing the PERx-capable functional group into peptides, we expect that the PERx principle can be similarly applied to generate peptide-based covalent radiopharmaceuticals.^44^ Moreover, covalent protein binders are able to cross-link both high and low-abundance targets efficiently.^45,46^ Unlike current low MW radioligands that are limited to highly overexpressed receptors, the covalent protein radiopharmaceuticals can be suitable for targets with various expression levels. Irreversible binding will also make covalent radiopharmaceuticals suitable for targets that do not internalize. Lastly, beyond cancer, the improved efficacy and safety of covalent protein radiopharmaceuticals will expand the scope of TRT to noncancerous diseases such as heart, gastrointestinal, endocrine and neurological diseases.

For the generalization of this covalent protein radiopharmaceutical strategy, the cross-linking kinetics and specificity are both critical. The reaction must be fast enough to cross-link sufficient targets before the drug clears the blood, and meanwhile must be target specific to avoid off-target cross-linking. The cross-linking kinetics can be affected by radiopharmaceutical and target concentration, their association and dissociation rate, as well as the reactivity between the Uaa and target residue. Therefore, selection of protein binder with appropriate binding kinetics, development of new latent bioreactive Uaas with enhanced proximity-enabled reactivity, and optimization of Uaa incorporation sites may facilitate the generation of effective covalent protein radiopharmaceuticals for various targets.^21^ In addition, pharmacokinetics differs between mice and humans, and our current study was performed in mice and did not address potential HER2 on-target toxicity, which both warrant further investigation for clinical translation.

In summary, covalent protein radiopharmaceuticals enabled highly specific, extended retention of radionuclide in tumors while sparing normal tissues, thus enhancing the efficacy and safety of TRT. Shifting the protein-based TRT from noncovalent to covalent binding mode, covalent protein radiopharmaceuticals have the potential to expand TRT across diverse targets and disease areas for precision medicine.

## Supporting Information Available

Experimental procedures for protein expression and purification, mass spectrometry, cross-linking of Nb_HER2_ with HER2 *in vitro*, on cells, and *in vivo*, radiolabeling of Nb_HER2_ with ^124^I and ^225^Ac, PET imaging in mice, and radiotherapy studies in mice. Supporting Figures S1–S6 and Supporting Tables 1–2 (PDF)
