# Supplementary material for: Covalent Proteins as Targeted Radionuclide Therapies Enhance Antitumor Effects
Source: ACS Cent Sci. 2023 Jun 7;9(6):1241–51. doi: 10.1021/acscentsci.3c00288 (PMC10311652; doi:10.1021/acscentsci.3c00288)
Supplement: Supplementary file 1 — oc3c00288_si_001.pdf [file oc3c00288_si_001.pdf]

Supporting Information

Covalent Proteins as Targeted Radionuclide Therapies Enhance Antitumor Effects

Paul C. Klauser[1,2], Shalini Chopra[1,2,3], Li Cao[1,2], Kondapa Naidu Bobba[3], Bingchen Yu[1,2], Youngho Seo[3], Emily Chan[4], Robert R. Flavell[1,2,3], Michael J. Evans[1, 2, 3], Lei Wang[1,2] *

1 Department of Pharmaceutical Chemistry and the Cardiovascular Research Institute, University of California San Francisco, San Francisco, California, 94158, USA.

2 Helen Diller Family Comprehensive Cancer Center, University of California San Francisco, San Francisco, 94158, USA

3 Department of Radiology and Biomedical Imaging, University of California San Francisco, San Francisco, 94158, USA

4 Department of Pathology, University of California San Francisco, San Francisco, 94158, USA

* Email: Lei.Wang2@ucsf.edu

Table of Contents

Supporting Experimental Procedures……………………………………………………S3
Supporting Figures…………………………………………………………………………S9
Supporting Tables…………………………………………………………………………S15

Supporting Experimental Procedures

Reagents

Primers were synthesized and purified by Integrated DNA Technologies (IDT), and plasmids were sequenced by GENEWIZ. All molecular biology reagents were either obtained from New England Biolabs or Vazyme. His-HRP and GAPDH-HRP antibodies were obtained from ProteinTech Group. HER2/ErbB2 antibody was obtained from Cell Signaling Technology.

Nb_{HER2} amino acid sequence

MKYLLPTAAAGLLLLAAQPAMAMKYLLPTAAAGLLLLAAQPAMAMGQVQLQESGGGSVQAGG
SLKLTCAASGYIFNSCGMGWYRQSPGRERELVSRISGDGDTWHKESVKGRFTISQDNVKKTLY
LQMNSLKPEDTAVYFCAVCYNLETYWGQGTQVTVSSHHHHHH

pelB leader sequence is highlighted in green.

Residue D54 in red was the site where FSY was incorporated.

Protein expression and purification

Plasmids pBad-Nb_{HER2} (WT) was transformed into SHuffle T7 Express electrocompetent *E. coli* cells. pBad-Nb_{HER2} (D54TAG) and pEvol-FSYRS was co-transformed DH10β electrocompetent *E. coli* cells. For expression, transformed bacteria was culture in 2xYT at 37 °C with either 100 μg/mL ampicillin only (for Nb_{HER2} (WT)) or 100 μg /mL ampicillin and 34 μg /mL chloramphenicol (for Nb_{HER2} (D54TAG)). The culture was induced with 0.2% arabinose once OD_{600} reached 0.6-0.8. For Nb_{HER2} (D54TAG), 1 mM FSY was added right before induction. The expression was induced for 16-20 h at 18 °C, and the bacterial pellets were collected by centrifugation at 8000 g for 15 min. The bacterial pellets were resuspended in lysis buffer (25 mM sodium phosphate, 500 mM NaCl, 20 mM imidazole, 1 mg/mL lysozyme, 0.1 mg/mL DNase, and protease inhibitor; pH 7.5). The IMAC chromatography was used for protein purification and the procedure was described previously.[1]

Mass spectrometry

Mass spectrometric measurements were performed as previously described.[1, 2] Mass spectra of intact proteins were obtained through electrospray ionization mass spectrometry, operating in positive electrospray ionization mode (ESI+), using a Xeno G25 Q-Tof mass spectrometer. The mass spectrometer was connected to an LC-20AD (Shimadzu) liquid chromatography unit. Using reverse phase chromatography, the protein sample was separate from small molecules using a

Waters Xbridge BEH C4 column (300 Å, 3.5 µm, 2.1 mm x 50 mm). An acetonitrile gradient (30-71.4%) with 0.1% formic acid was used with a 0.2 mL/min constant flow rate at RT. Data was obtained at a rate of 1 sec/scan between 10000 to 18000 m/z. MassLynx mass spectrometry software was used to deconvolute the spectra. Nb_{HER2} mass was calculated with the pelB leader sequence removed and the formation of two disulfide bonds.

For tandem mass spectrometry, the peptides were analyzed and sequence using a Q Exactive Orbitrap interfaced with Ultimate 3000 LC system. To prepare the peptide, 5 μg of Nb_{HER2} (FSY) was incubated with 12.5 μg of HER2 ECD for 16 h at 37 °C. The cross-linking reaction was then subsequently digested with trypsin. The trypsin digested cross-link peptide was then injected into an Ace UltraCore super C18 reverse-phase column (300 Å, 2.5 µm, 75 mm x 2.1 mm). An acetonitrile gradient (5-95%) with 0.1% formic acid was used with a 0.2 mL/min constant flow rate at RT. Stepped collision energy HCD was used to fragmentize the cross-linked peptide with a normalized collision energy of 28, 30, 35 eV. Survey scans were acquired at a resolution of 70,000 at m/z 200 on the Q Exactive. Cross-linked peptides were searched with pLink 2 and OpenUaa search engine.[3, 4]

Cross-linking of Nb_{HER2} with HER2 ECD *in vitro*

Recombinant extra-cellular domain (ECD) of HER2 receptor was purchased from Abcam (Cat# ab168896). Purified 1 μM Nb_{HER2} (WT) or Nb_{HER2} (FSY) was incubated with 1 μM HER2 in 10 uL 1 X PBS, pH 7.4 for 4 h or indicated time points at 37 °C. After incubation, 4x Laemmli sample buffer (Bio Rad, Cat# 161-0747) with 2-mercaptoethanol was added into the incubation and heated at 95 °C for 10 min. The samples were separated on SDS-PAGE and either analyzed by Coomassie blue staining or Western blot. For Western blotting, the PVDF membrane was blocked with 5% milk for 1 h at RT while rocking. The membrane was then treated with 1:10000 anti-his monoclonal antibody (Proteintech #HRP66005) in 5% milk at RT while rocking. The membrane was then washed three times with PBST before imaging.

Biolayer interferometry (BLI) measurement

The association kinetics between human HER2 and Nb_{HER2} was measured with BLI using the Octet Red384 system (ForteBio). Biotinylated human HER2 protein (HE2-H82E2, Acro Biosystems) was first loaded to streptavidin (SA) sensor (ForteBio #18-5019) by incubating the SA sensor in 100 nM biotinylated human HER2 in the Kinetic Buffer (0.005 % (v/v) Tween 20 and 0.1 % BSA in PBS, pH = 7.4) at 25 °C. The sensor was equilibrated (baseline step) in the Kinetic

Buffer for 120 s, after which the sensor was incubated with varying concentrations (12.5, 25, 50, 100, and 200 nM) of Nb_{HER2}(WT) or Nb_{HER2}(FSY) (association step) for 90 s, followed with dissociation step in the Kinetic Buffer for 400 s. Data was fitted for a 1:1 stoichiometry and kinetic rate constant was calculated using the built-in software.

Cross-linking of Nb_{HER2} with HER2 on cells

NCI-N87 cells ($2x10^5$) was seeded in 12-well plates and cultured with RPMI 1640 + 10% FBS. After 16 h, 1 μM Nb_{HER2} (WT) or Nb_{HER2} (FSY) was added into culture for a final 0.5 mL volume. After 3 h incubation, the cells were washed twice with 1X PBS and dissociated with enzyme free dissociation buffer. The cells were collected and lysed with 100 uL Pierce RIPA buffer with protease inhibitor cocktail for 1 h on ice. The cell lysates were analyzed with Western blots using antibodies specific for Hisx6 (Proteintech #HRP66005, 1:10000 dilution), HER2 (Cell signaling #2165S 1:1000 dilution), or GAPDH (Proteintech #HRP60004, 1:10000 dilution). The Western blots detecting HER2 required a secondary anti-Rabbit incubation (scbiotech #sc-2357, 1:5000 dilution). The Western blot condition is described above.

Cross-linking of Nb_{HER2} with HER2 on tumor in vivo

NCI-N87 cells (5.0×10^6) were resuspended in 200 μL PBS and subcutaneously injected into the left flank in mice (age 6-8 weeks, male, 25.0 ± 3.0 g). After 21 days, 50 μg of either Nb_{HER2} (WT) or Nb_{HER2} (FSY) and 30 mg L-lysine was intravenously injected into each mouse. The mice were sacrificed at 6 h post-injection and the NCI-N87 tumor was excised from the mice. The tumors were added 500 μL of RIPA and 1x protease inhibitor and was homogenized and lysed. The cell lysates were analyzed with Western blot as described above.

^{124}I Radiolabeling of Nb_{HER2}

Nb_{HER2} (WT) or Nb_{HER2} (FSY) was labeled with ^{124}I by using the direct iodination strategy. In a Pierce iodination tube, 3.0 mCi of Na^{124}I was added and the pH was adjusted to 7 with 100 μL HEPES (1 M). Three mg of Nb_{HER2} (WT) or Nb_{HER2} (FSY) were added to the iodination tube and the reaction was carried out for 20 min at room temperature with frequent shaking. To check the radiolabeling efficiency, instant thin layer chromatography (iTLC) was performed using Whatman filter paper and 20 mM citric acid as mobile phase. The radiolabeled proteins were purified using G25 columns and PBS.

Preparation of Macropa-PEG$_4$-Nb$_{HER2}$ and ^{225}Ac radiolabeling

Macropa-PEG$_4$-TFP ester was synthesized using a previously reported method.[5] 110 nmol of Macropa-PEG$_4$-TFP ester was incubated with 7 nmol of either Nb$_{HER2}$ (WT) or Nb$_{HER2}$ (FSY) in 0.1 M Carbonate-Bicarbonate buffer pH 9.0 at 37 °C for 2 h. Then, Macropa-PEG$_4$-Nb$_{HER2}$ (WT) or Macropa-PEG$_4$-Nb$_{HER2}$ (FSY) was purified with a PD-10 desalting column using PBS, pH 7.4 as an eluent. For radiolabeling, actinium-225 (10 μCi) was incubated with either 15 pmol Macropa-PEG$_4$-Nb$_{HER2}$ (WT) or 15 pmol Macropa-PEG$_4$-Nb$_{HER2}$ (FSY) in 20 μL 2M NH_4OAc pH 5.8 buffer and 5 μL of L-Ascobic Acid (150 mg/mL). The reaction proceeded for 30 min at 37 °C while shaking. The radiolabeling efficiency was measured using iTLC using iTLC-SG using 10 mM EDTA, pH=5.5 as a mobile phase and the radiolabeling yields were > ~95%. The radioimmunoconjugates ^{225}Ac-Nb$_{HER2}$(WT) and ^{225}Ac-Nb$_{HER2}$(FSY) were injected into mice without further purification.

Mice

All mouse protocols were approved by institutional animal care and use program and were carried out in compliance with the guidelines published by the Association for Assessment and Accreditation of Laboratory Animal Care (AAALAC). Mice were randomly assigned to experimental groups. Male mice 6-8 weeks, Nu/J (stain# 002019, The Jackson Laboratory) were used in all *in vivo* studies.

In vivo ^{124}I-Nb$_{HER2}$(WT) and ^{124}I-Nb$_{HER2}$(FSY) PET imaging studies

PET imaging with ^{124}I labeled Nb$_{HER2}$ (WT) or Nb$_{HER2}$ (FSY) were done in NCI-N87 tumor bearing nude mice (age 6-8 weeks, male, 25.0 ± 3.0 g). NCI-N87 tumors were xenografted in mice by injecting 5.0×10^6 NCI-N87 cells subcutaneously in left flank. The mice were injected intravenously with 0.25 mg of cold NaI to suppress thyroid uptake. The kidney uptake was suppressed by co-injection of 30 mg of L-lysine per mice. Mice were injected with 50.0 ± 5.0 μCi ^{124}I labeled nanobodies (n = 4 for Nb$_{HER2}$ (FSY), n = 3 for Nb$_{HER2}$ (WT)) for the dynamic PET imaging and 80.0 – 100.0 μCi for the static PET imaging intravenously. PET dynamic images were acquired for 90 min with CT for 5 min. Static PET/CT images (n = 4 for Nb$_{HER2}$ (FSY), n = 3 for Nb$_{HER2}$ (WT)) were acquired at different time points post injections (3 h, 6 h,10 h, 24 h, 48 h, 72 h). PET data were

acquired on Seimens Inveon microPET/CT. The PET data was reconstructed and analyzed with AMIDE software.

In vivo ^{225}Ac-Nb$_{HER2}$(WT) and ^{225}Ac-Nb$_{HER2}$(FSY) therapy studies

NCI-N87 tumors were xenografted in mice as described above. Once the xenografted-tumor reached 100-300 mm^3 (Day 0), the mice were injected with 0.8 ± 0.2 μC ^{225}Ac labeled nanobodies (n = 8 for Nb$_{HER2}$ (FSY), n = 7 for Nb$_{HER2}$ (WT)) or Saline (n=5). The mice were also coinjected with 30 mg of L-lysine per mice. On Day 7, the mice were coinjected again with 0.8 ± 0.2 μC ^{225}Ac labeled nanobodies or saline and 30 mg of L-lysine per mice. The tumor growth was with a digital caliper in two dimensions, and tumor volume was calculated using the formula: tumor volume = length x width^2/2. At day 26, the mice were sacrificed for analysis. The liver, kidney, heart, and bone were dissected and processed for hematoxylin and eosin (H&E) staining and analysis. The microscopic images of the H&E slides were analyzed by a trained pathologist (E.C.) and no abnormalities were observed with the tissues.

Quantification and statistical analysis

All quantitative data and statistical analysis were analyzed by Student's t test using Prism 6.0 (GraphPad software). All the P values were calculated using GraphPad PRISM 6.0 with the following significance: n.s. $p > 0.05$; * $p < 0.05$; ** $p < 0.01$; *** $p < 0.001$; **** $p < 0.0001$. Statistical details for each experiment can be found in the figures and the legends.

Supporting References

1. Liu, J., Li, S., Aslam, N.A., Zheng, F., Yang, B., Cheng, R., Wang, N., Rozovsky, S., Wang, P.G., Wang, Q., et al. Genetically Encoding Photocaged Quinone Methide to Multitarget Protein Residues Covalently in Vivo. *J. Am. Chem. Soc.* **2019**, *141*, 9458–9462. DOI: 10.1021/jacs.9b01738.

2. Liu, J., Zheng, F., Cheng, R., Li, S., Rozovsky, S., Wang, Q., and Wang, L. Site-Specific Incorporation of Selenocysteine Using an Expanded Genetic Code and Palladium-Mediated Chemical Deprotection. *J. Am. Chem. Soc.* **2018**,*140*, 8807–8816. DOI: 10.1021/jacs.8b04603.

3. Chen, Z.-L., Meng, J.-M., Cao, Y., Yin, J.-L., Fang, R.-Q., Fan, S.-B., Liu, C., Zeng, W.-F., Ding, Y.-H., Tan, D., et al. A high-speed search engine pLink 2 with systematic evaluation for

proteome-scale identification of cross-linked peptides. *Nat. Commun.* **2019**, *10*, 3404. DOI: 10.1038/s41467-019-11337-z.

4. Liu, C., Wu, T., Shu, X., Li, S., Wang, D.R., Wang, N., Zhou, R., Yang, H., Jiang, H., Hendriks, I.A., et al. Identification of Protein Direct Interactome with Genetic Code Expansion and Search Engine OpenUaa. *Adv. Biology* **2021**, *5*, 2000308. DOI: 10.1002/adbi.202000308.

5. Bobba, K. N.; Bidkar, A. P.; Meher, N.; Fong, C.; Wadhwa, A.; Dhrona, S.; Sorlin, A.; Bidlingmaier, S.; Shuere, B.; He, J.; Wilson, D. M.; Liu, B.; Seo, Y.; VanBrocklin, H. F.; Flavell, R. R. Evaluation of ^{134}Ce/^{134}La as a PET Imaging Theranostic Pair for ^{225}Ac α-Radiotherapeutics. *J. Nucl. Med.* **2023**, in press https://doi.org/10.2967/jnumed.122.265355.

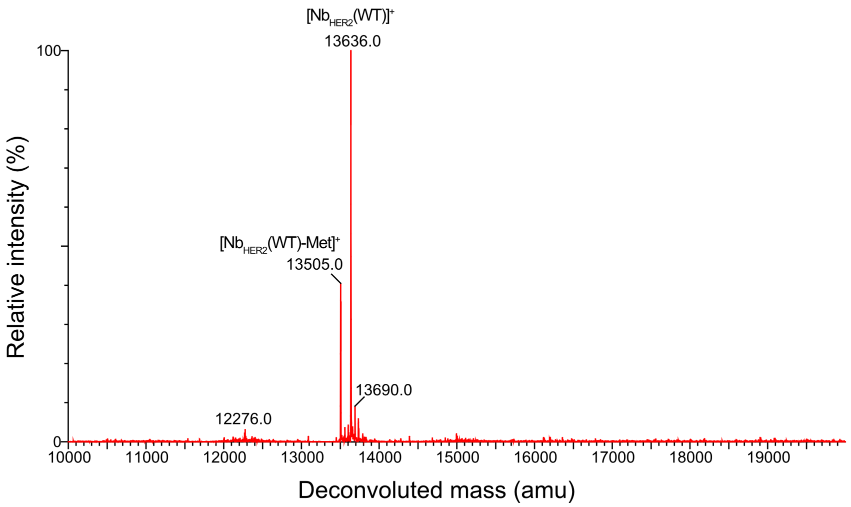

Figure S1. Mass Spectrum of Nb$_{HER2}$(WT). Electrospray ionization time-of-flight mass spectrum of intact Nb$_{HER2}$(WT). Expected mass 13636 Da; observed 13636 Da. The -Met peak was also detected, which indicates loss of N-terminal Met residue and is expected for proteins expressed in *E. coli* cells.

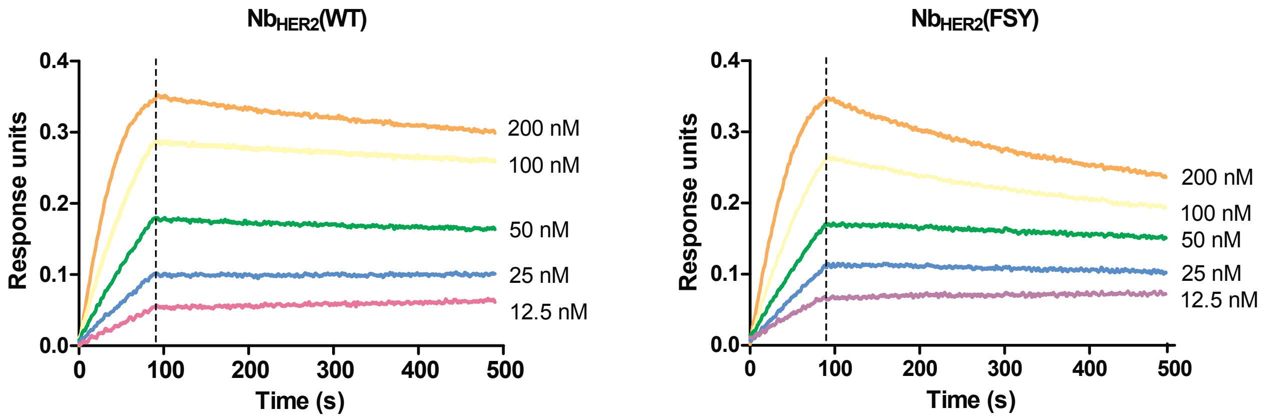

Figure S2. Biolayer interferometry of HER2 and Nb$_{HER2}$(WT) or Nb$_{HER2}$(FSY). Biotinylated human HER2 was loaded onto the streptavidin sensor and then incubated with varying concentrations of Nb$_{HER2}$(WT) or Nb$_{HER2}$(FSY) for 90 seconds followed with dissociation for 400 seconds.

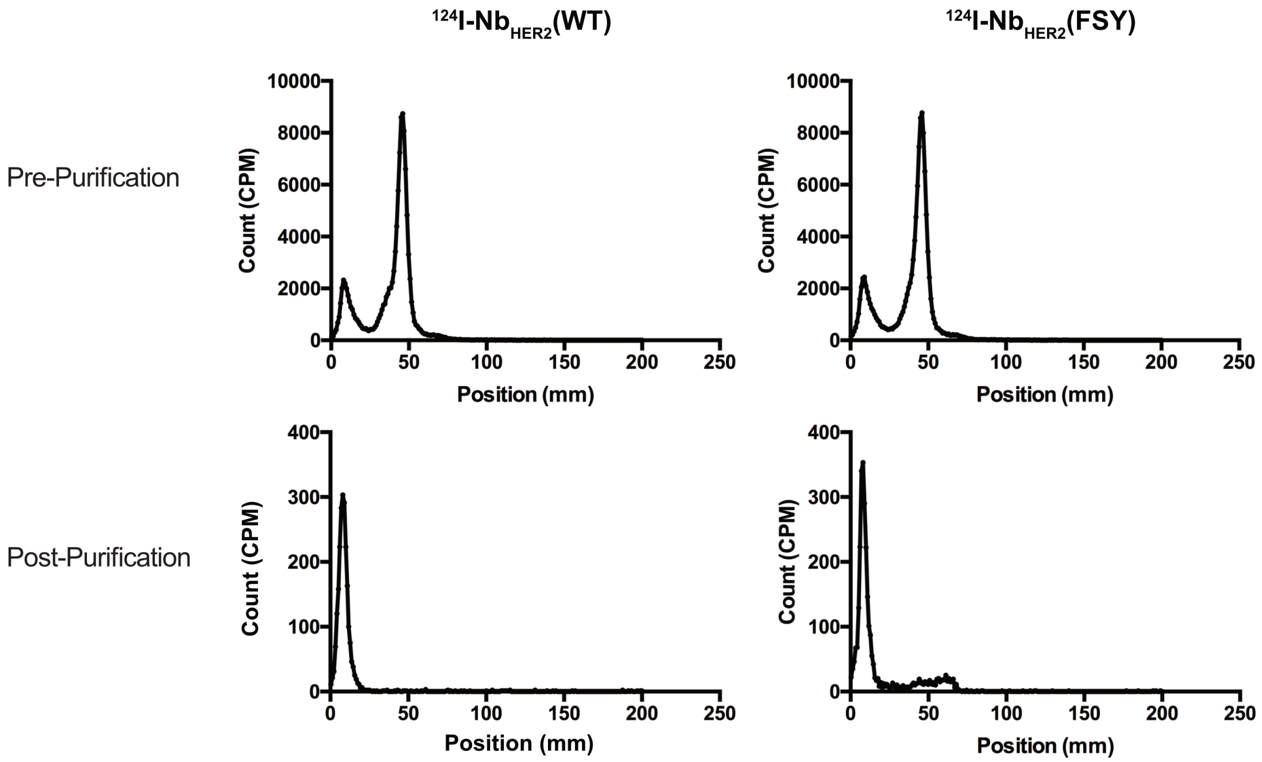

Figure S3. Radiochemical yield and purity of ^{124}I-Nb$_{HER2}$(WT) and ^{124}I-Nb$_{HER2}$(FSY). Radiolabeling efficiency for ^{124}I-Nb$_{HER2}$(WT) and ^{124}I-Nb$_{HER2}$(FSY) measured though iTLC. The calculated radiochemical yield was 24.5% for ^{124}I-Nb$_{HER2}$(WT) and 23.5% for ^{124}I-Nb$_{HER2}$(FSY). The calculated post-purification radiochemical purity was 99.9% for ^{124}I-Nb$_{HER2}$(WT) and 95.2% for ^{124}I-Nb$_{HER2}$(FSY).

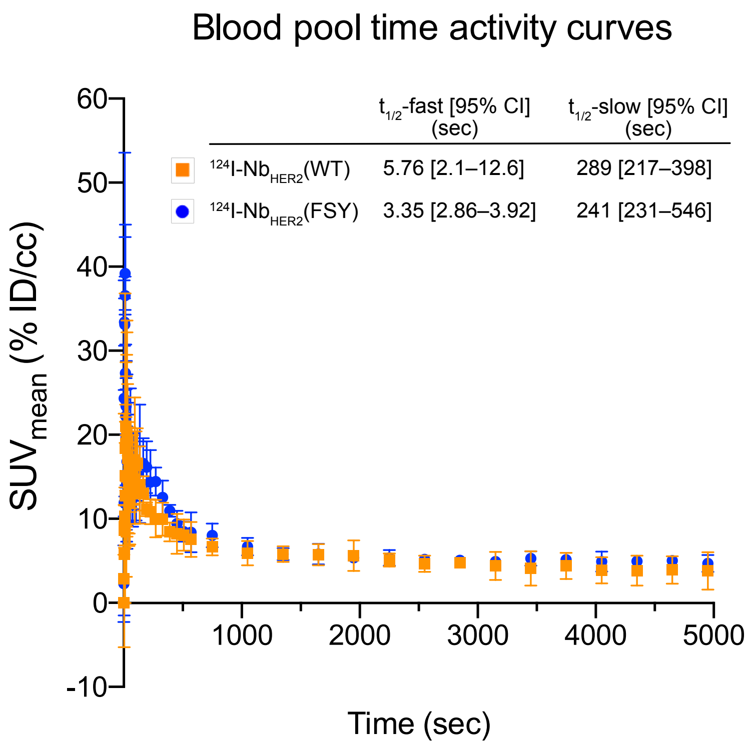

Figure S4. ^{124}**I-Nb**$_{\mathbf{HER2}}$**(WT) and** ^{124}**I-Nb**$_{\mathbf{HER2}}$**(FSY) both were cleared from blood rapidly.** Dynamic PET acquisition was used to measure the standardized uptake value (SUV) in blood in percent injected dose per cm^3 (% ID/cc) at indicated time points. The data were fitted with two phase exponential decay to yield the $t_{1/2}$ values. Error bars represent 95% CI; n = 3 mice.

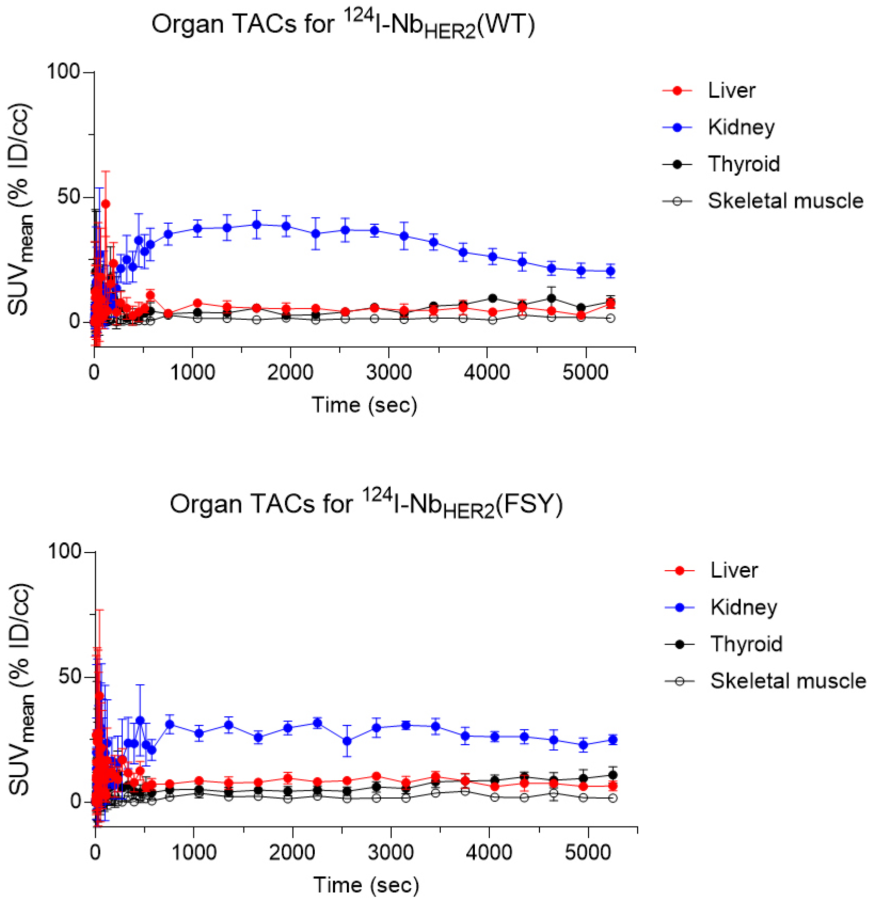

Figure S5. ^{124}I-Nb$_{HER2}$(WT) and ^{124}I-Nb$_{HER2}$(FSY) showed similar biodistribution in normal organs. Standardized uptake value (SUV) in percent injected dose per cm^3 (% ID/cc) was plotted with time to yield the time activity curve (TAC) in indicated organs. Error bars represent s.d.; n = 3 mice for ^{124}I-Nb$_{HER2}$(WT) injection; n = 4 mice for ^{124}I-Nb$_{HER2}$(FSY) injection.

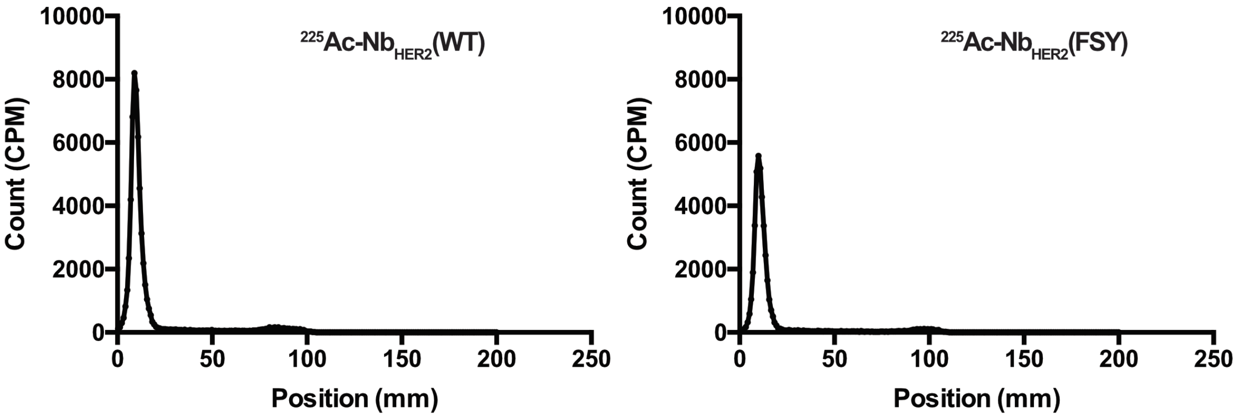

Figure S6. Radiochemical yield of ^{225}Ac-Nb$_{HER2}$(WT) and ^{225}Ac-Nb$_{HER2}$(FSY). Radiolabeling efficiency for ^{225}Ac-Nb$_{HER2}$(WT) and ^{225}Ac-Nb$_{HER2}$(FSY) measured though iTLC. The calculated radiochemical yield was 96.6% for ^{225}Ac-Nb$_{HER2}$(WT) and 95.5% for ^{225}Ac-Nb$_{HER2}$(FSY).

Supporting Table 1. Primers used for cloning Nb_{HER2}(D54TAG)

Primer	Oligonucleotide Sequence (5'→3')
Nb_{HER2}-54TAG-For	TCGTATCTCCGGCTAGGGCGACACGTGGCATAAAGA
Nb_{HER2}-54TAG-Rev	CCTAGCCGGAGATACGAGAAACCAGCTCGCGC

Supporting Table 2. Values in Figure S3.

Time	^{124}I-Nb$_{HER2}$(WT)			^{124}I-Nb$_{HER2}$(FSY)		
(Sec)	Mean	%CV	n	Mean	%CV	n
0.0	0.000	0.000	3	0.000	0.000	3
1.0	2.873	39.809	3	2.273	81.006	3
3.0	5.791	40.821	3	11.922	45.349	3
5.0	8.633	64.796	3	24.338	23.252	3
7.0	9.079	11.151	3	33.405	3.384	3
9.0	10.302	13.451	3	33.105	24.861	3
11.0	12.786	19.769	3	39.200	4.458	3
13.0	18.398	40.322	3	36.570	2.479	3
15.0	15.121	17.214	3	27.334	26.049	3
17.0	18.467	11.206	3	22.210	15.471	3
19.0	18.945	20.587	3	23.417	5.760	3
21.0	21.018	2.105	3	19.693	11.706	3
23.0	15.043	31.929	3	18.727	21.577	3
25.0	19.299	21.352	3	14.158	19.489	3
27.0	20.209	26.688	3	15.468	6.286	3
29.0	20.038	24.442	3	16.823	24.845	3
32.5	17.167	20.796	3	15.108	10.015	3
37.5	14.845	7.211	3	18.160	12.574	3
42.5	14.144	20.985	3	17.407	11.565	3
47.5	11.993	12.520	3	15.046	6.898	3
52.5	13.925	20.116	3	14.842	5.571	3
57.5	14.976	11.220	3	17.534	18.316	3
65.0	14.689	9.445	3	16.528	7.026	3
75.0	15.824	10.572	3	14.723	15.565	3
85.0	14.692	7.968	3	15.665	11.620	3
95.0	16.917	17.906	3	16.121	7.816	3
105.0	16.416	9.846	3	14.200	12.561	3
115.0	16.608	10.107	3	15.408	8.527	3
135.0	14.019	13.281	3	16.551	17.095	3
165.0	13.020	6.328	3	16.571	7.342	3
195.0	11.143	2.927	3	16.144	7.633	3
225.0	10.826	5.637	3	14.381	10.723	3
270.0	10.028	8.974	3	14.438	4.609	3
330.0	9.955	7.951	3	12.543	6.453	3
390.0	8.439	5.362	3	10.963	2.613	3
450.0	8.209	11.667	3	9.431	6.445	3
510.0	8.047	9.336	3	8.470	5.733	3
570.0	7.550	11.109	3	8.389	11.446	3
750.0	6.671	6.013	3	8.025	7.158	3
1050.0	5.930	9.837	3	6.680	6.330	3
1350.0	5.788	6.635	3	5.802	4.882	3
1650.0	5.729	8.825	3	5.787	8.566	3
1950.0	5.625	13.164	3	5.359	4.329	3
2250.0	5.111	6.317	3	5.365	7.036	3
2550.0	4.660	8.224	3	5.163	4.561	3
2850.0	4.766	4.851	3	5.074	5.178	3
3150.0	4.382	15.335	3	4.912	3.419	3
3450.0	4.092	20.182	3	5.280	6.493	3
3750.0	4.410	14.264	3	5.124	1.424	3
4050.0	3.869	16.361	3	4.910	9.841	3
4350.0	3.856	18.892	3	4.954	2.017	3
4650.0	3.911	16.928	3	5.040	1.697	3
4950.0	3.801	23.464	3	4.693	8.517	3
